# Comparative study of ciprofol vs. propofol in carotid endarterectomy: focusing on mean arterial pressure, vasoactive drug use, and postoperative complications

**DOI:** 10.3389/fmed.2025.1648417

**Published:** 2025-10-17

**Authors:** Xinxin Zheng, Shiyun Deng, Lu Tian, Yanqing Zhang, Lin Bai

**Affiliations:** ^1^Department of Anesthesiology, University-Town Hospital, Chongqing Medical University, Chongqing, China; ^2^Department of Anesthesiology, Children’s Hospital, Chongqing Medical University, Chongqing, China

**Keywords:** ciprofol, propofol, anesthesia, mean arterial pressure, carotid endarterectomy

## Abstract

**Aim:**

The aim of this study was to compare the effects of ciprofol and propofol on mean arterial pressure (MAP) management, vasoactive drug usage, and postoperative complications after carotid endarterectomy.

**Methods:**

A total of 103 patients were included in either the ciprofol (*n* = 50) or propofol (*n* = 53) group. The MAP was recorded at nine perioperative timepoints from before anesthesia (T0) to extubation (T8). We focused on the achievement rate of the target MAP during carotid cross-clamping at T4–T6. We also examined vasopressor use (norepinephrine, urapidil) and postoperative complications.

**Results:**

In terms of primary outcomes, the ciprofol group exhibited a higher MAP at T3 (before the skin incision; *p* = 0.006) and achieved the target MAP faster at T4–T5 (during carotid cross-clamping and 5 min after cross-clamping; *p* < 0.001) than did the propofol group. There were no statistically significant differences between the groups at T6 (10 min after cross-clamping; *p* = 0.360). The ciprofol group exhibited significantly better hemodynamic stability during extubation (*p* < 0.001). Regarding the secondary outcomes, the ciprofol group was administered a lower dosage of norepinephrine (*p* < 0.001) and had fewer cases of early cognitive dysfunction (eCD) (*p* = 0.024).

**Conclusion:**

These findings suggest that ciprofol offers advantages over propofol during carotid endarterectomy by optimizing MAP control, minimizing vasopressor use, and mitigating postoperative complications. Ciprofol may be the preferable anesthetic agent in carotid artery-related procedures.

**Clinical trial registration:**

https://www.chictr.org.cn/, identifier ChiCTR2500104162.

## 1 Introduction

The prevalence of carotid stenosis is increasing each year worldwide (1). CEA is the primary treatment for carotid stenosis and indicated for patients with more than 70% stenosis, resulting in a 50% reduced risk of stroke (2, 3). During the carotid cross-clamping phase of CEA, the mean arterial pressure (MAP) should be more than 20% higher than the baseline value following the administration of vasopressors (4, 5). This helps maintain the cerebral perfusion pressure and reduces the risks of early cognitive dysfunction (eCD), cerebral ischemia, and perioperative complications (6). To achieve the target MAP, vasopressor intervention is often needed (7), with norepinephrine being the most commonly used vasoactive drug. However, studies have revealed that after carotid artery reperfusion, a MAP more than 40% above the baseline value or reaching 125 mmHg may increase the risk of cerebral hyperperfusion syndrome, leading to headaches (8).

Propofol is a widely used anesthetic agent. However, propofol can cause myocardial depression and peripheral vasodilation. This often results in a decreased MAP (9). Ciprofol (2,6-disubstituted phenol analog), an improved version of propofol, retains the advantages of propofol, such as rapid onset, short half-life, and quick recovery, while also featuring minimal respiratory depression and slight injection-site pain. Numerous studies on sedation for gastrointestinal endoscopy, fiberoptic bronchoscopy, general anesthesia induction, and critical care medicine have demonstrated the safety and efficacy of ciprofol (10–12). However, research on the hemodynamic stability of special populations (such as neurosurgical and cardiothoracic surgical patients) receiving ciprofol remains limited, particularly concerning MAP management during carotid endarterectomy (CEA).

Inadequate MAP control is a key risk factor for stroke and perioperative mortality in CEA patients (13, 14). Therefore, selecting appropriate anesthetic agents and using an appropriate amount of vasopressors are highly important for perioperative MAP management during CEA.

## 2 Materials and methods

### 2.1 Patient population

This study was approved by the Ethics Committee of University-Town Hospital of Chongqing Medical University (IIT-LL-2025067) and was registered in the Chinese Clinical Trials Registry (ChiCTR2500104162). Clinical data from 103 patients who underwent carotid endarterectomy between 1 January 2023 and 31 December 2024 were collected. The inclusion criteria included an American Society of Anesthesiologists (ASA) physical status classification of II–III, a body mass index (BMI) between 18 and 30, and a preoperative MAP below 107 mmHg (140/90 mmHg). The exclusion criteria included incomplete perioperative data records or missing postoperative follow-up data; long-term use of sedatives or analgesics before surgery; occurrence of severe anaphylactic shock, pulmonary embolism, or other adverse events unrelated to the study drugs during anesthesia; placement of a vascular shunt during carotid cross-clamping; and bispectral index (BIS) values outside the range of 40–60 during surgery. On the basis of these criteria, 50 patients were included in the ciprofol group, and 53 patients were included in the propofol group.

### 2.2 Procedure

This was a single-center retrospective cohort study. A standardized protocol for anesthesia induction, maintenance, and extubation was used for all the patients included in the study. Upon entering the operating room, the medical team performs invasive radial artery blood pressure monitoring for each patient, records the BIS every 15 min and continuously monitors regional cerebral oxygen saturation (rSO2).

The standardized induction protocol used in the department was as follows: patients were preoxygenated with 100% high-flow oxygen, and then administered intravenous midazolam (2–4 mg/kg), sufentanil (0.5 μg/kg), and either ciprofol (50 mg 20 mL, H20200013, Liaoning Haisco Pharmaceutical Co., Ltd.) at 0.4 mg/kg (ciprofol group) or propofol (200 mg 20 mL, H20171278, ASPEN Pharmaceutical Co., Ltd.) at 2 mg/kg (propofol group). After the BIS value dropped below 60, cisatracurium besylate (0.15 mg/kg) was administered intravenously as a neuromuscular blocking agent for tracheal intubation. Anesthesia was maintained with intravenous infusions of remifentanil (10–15 μg/kg/h) and cisatracurium besylate (0.05 mg/kg/h). For sedation maintenance, ciprofol was infused at 1–2 mg/kg/h in the ciprofol group, and propofol was infused at 4–8 mg/kg in the propofol group. By excluding patients with BIS values outside the 40–60 range, we can minimize the confounding effects of sedation depth on MAP and vasoactive drug usage. When cerebral oxygen saturation dropped below 50%, the surgeon placed a vascular shunt. When the MAP exceeded 120 mmHg, urapidil was typically administered to manage hypertension.

### 2.3 Data collection

Data regarding the anesthetic agents and intraoperative events were continuously recorded using the MEDICALSYSTEM (Suzhou Medical System Technology Co., Ltd.). We extracted data at the following time points for both groups: T0 (before anesthesia induction), T1 (after anesthesia induction), T2 (during endotracheal intubation), T3 (before skin incision), T4 (during cross-clamping of the carotid artery), T5 (5 min after cross-clamping), T6 (10 min after cross-clamping), T7 (5 min after carotid artery reperfusion), and T8 (during endotracheal extubation). The primary outcomes included the MAP at T1–T8 and the rate at which the target MAP was reached during cross-clamping (T0 was the baseline MAP, and the target MAP was defined as an MAP more than 20% higher than the baseline MAP). The secondary outcomes included the use of vasoactive drugs (norepinephrine and urapidil) and postoperative complications. Data on eCD and headache within 24 h postoperatively were obtained from anesthesia postoperative follow-up records during hospitalization. The Montreal Cognitive Assessment (MoCA) was used to assess postoperative cognitive function in these patients; a score <26 was defined as indicative of eCD (15, 16). Postoperative complications (stroke, myocardial infarction, death) within 30 days were recorded through telephone follow-up. Additionally, anesthesia time, surgical time, carotid cross-clamping time, net fluid intake and the use of midazolam, sufentanil, remifentanil, and cisatracurium besylate throughout the anesthesia period were also recorded.

### 2.4 Statistical analysis

Statistical analyses were conducted using R (4.3.2). Continuous variables are presented as means (standard deviations, SDs) or medians (interquartile ranges, IQRs), depending on the distribution of the data. Categorical variables are described as frequencies and percentages (*n*%). To compare continuous variables between two independent groups, the Wilcoxon rank sum test was used for nonnormally distributed data. For categorical variables, Pearson’s chi-square test was applied when the expected count in each cell was five or more. In cases where the expected count was less than five, Fisher’s exact test was used instead. Multivariable analysis was performed using a linear regression model to assess the association between the dependent variable and multiple independent variables, adjusting for potential confounders. The level of significance was set at a *p* value < 0.05 for all tests.

To evaluate potential bias from excluded patients, we compared demographics between included and excluded cases using the Wilcoxon rank sum test, the chi-square test and Fisher’s exact test as appropriate. The results are detailed in Supplementary Table S1.

### 2.5 Power calculation

*Post hoc* power analysis indicated 80% power to detect a 15% difference in MAP achievement rates (*α* = 0.05).

## 3 Results

**Figure 1:**
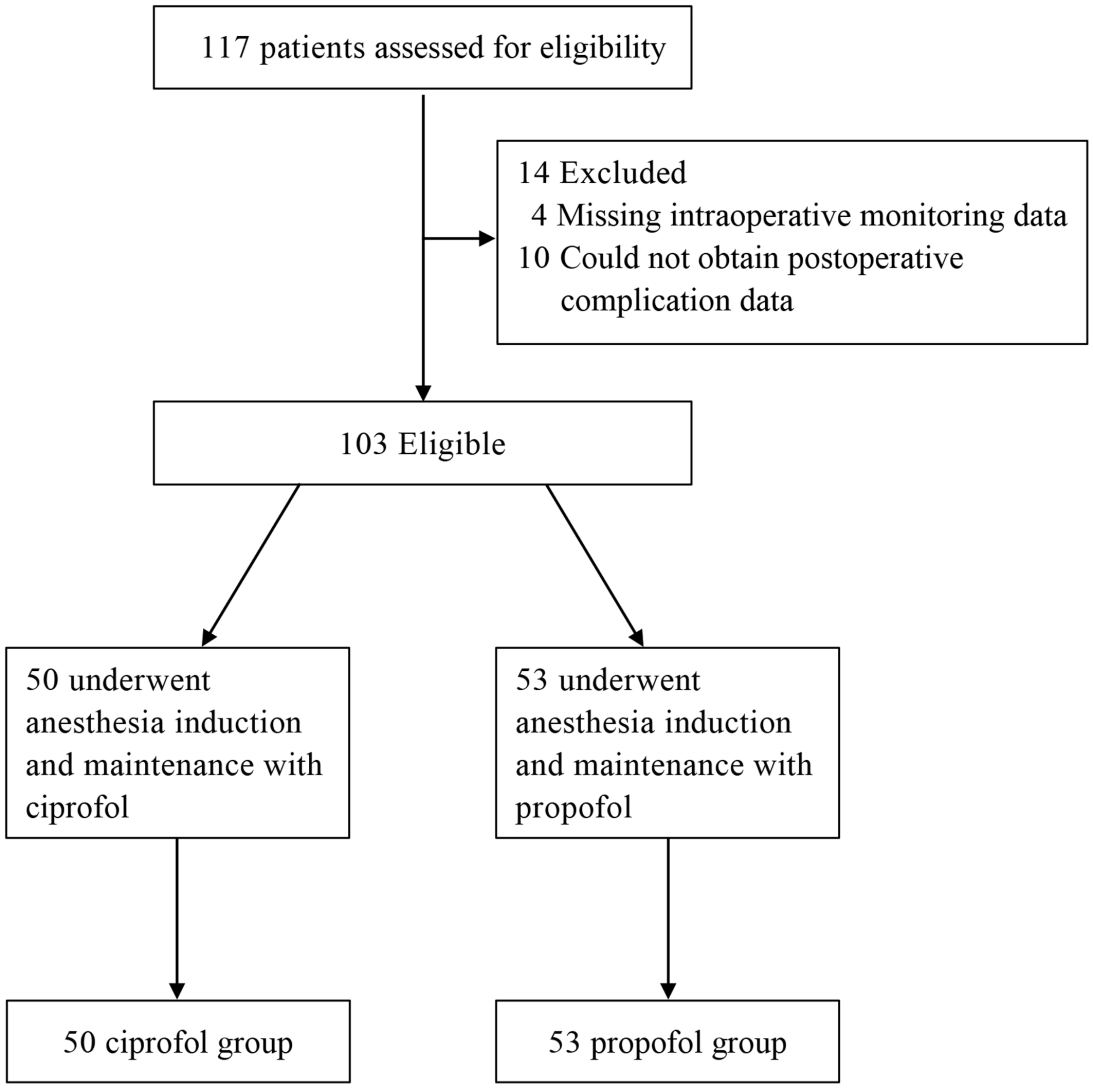
STROBE flow diagram.

A total of 117 patients were assessed for eligibility; however, four patients were excluded because of missing intraoperative monitoring data, and 10 were excluded because of our inability to obtain postoperative complication information during the telephone follow-up. One hundred three patients were ultimately eligible and grouped on the basis of the anesthetic agents used during the procedure. Fifty patients were assigned to the ciprofol group, and 53 patients were assigned to the propofol group (Figure 1). The basic demographic characteristics of the patients were similar between the two groups (Table 1).

**Table 1:** Demographics and baseline characteristics.

Characteristic	Overall, *N* = 103^a^	Ciprofol, *N* = 50^a^	Propofol, *N* = 53^a^	*p* value^b^
Age	71.0 (66.0, 75.0)	70.0 (65.0, 74.0)	71.0 (67.0, 76.0)	0.350
Sex				0.973
Female	29 (28.2%)	14 (28.0%)	15 (28.3%)	
Male	74 (71.8%)	36 (72.0%)	38 (71.7%)	
BMI	25.0 (23.0, 26.0)	24.5 (22.3, 26.0)	25.0 (23.0, 26.0)	0.479
ASA				0.402
II	31 (30.1%)	17 (34.0%)	14 (26.4%)	
III	72 (69.9%)	33 (66.0%)	39 (73.6%)	
Smoke				0.443
Yes	41 (39.8%)	18 (36.0%)	23 (43.4%)	
No	62 (60.2%)	32 (64.0%)	30 (56.6%)	
Hypertension				0.211
Yes	60 (58.3%)	26 (52.0%)	34 (64.2%)	
No	43 (41.7%)	24 (48.0%)	19 (35.8%)	
DM				0.616
Yes	50 (48.5%)	23 (46.0%)	27 (50.9%)	
No	53 (51.5%)	27 (54.0%)	26 (49.1%)	
CHD				0.142
Yes	34 (33.0%)	13 (26.0%)	21 (39.6%)	
No	69 (67.0%)	37 (74.0%)	32 (60.4%)	
CVA history				0.983
Yes	31 (30.1%)	15 (30.0%)	16 (30.2%)	
No	72 (69.9%)	35 (70.0%)	37 (69.8%)	

a Median (IQR); *n* (%).

b Wilcoxon rank sum test; Pearson’s Chi-squared test.

BMI, body mass index; ASA, American Society of Anesthesiologist; DM, Diabetes Mellitus; CHD, Coronary Heart Disease; CVA history, history of cerebrovascular accident.

### 3.1 MAP management parameters

**Figure 2:**
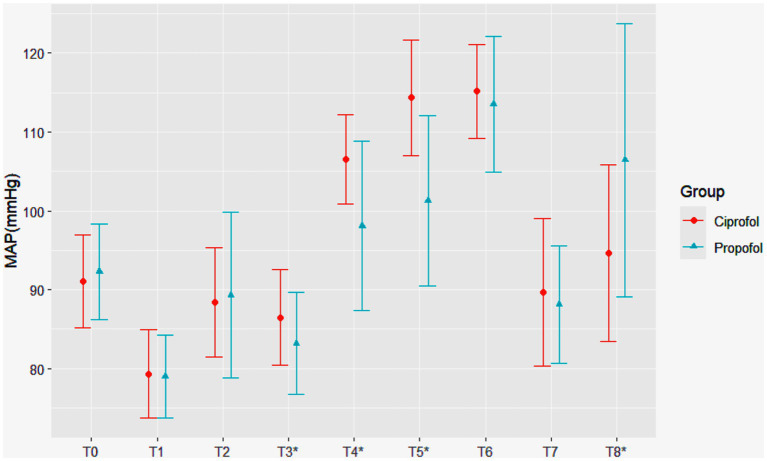
Changes in mean arterial pressure (MAP) over time in patients receiving ciprofol and propofol. Statistically significant hemodynamic differences were observed at key timepoints: before the skin incision (T3: 86.5 ± 6.1 mmHg vs. 83.2 ± 6.5 mmHg, *p* = 0.006), carotid cross-clamping (T4: 106.5 ± 5.6 mmHg vs. 98.1 ± 10.8 mmHg, *p* < 0.001), 5 min after cross-clamping (T5: 114.4 ± 7.3 mmHg vs. 101.3 ± 10.8 mmHg, *p* < 0.001), and endotracheal extubation (T8: 94.6 ± 11.2 mmHg vs. 106.4 ± 17.3 mmHg, *p* < 0.001), where asterisks denote Statistically significant difference. The ciprofol and propofol groups are represented by red circles and blue triangles, respectively.

In terms of the primary outcomes, the ciprofol group had higher MAP at T3 (86.5 ± 6.1 mmHg vs. 83.2 ± 6.5 mmHg, *p* = 0.006). During the early period of cross-clamping, the differences between the two groups were significant at T4 (106.5 ± 5.6 mmHg vs. 98.1 ± 10.8 mmHg, *p* < 0.001) and T5 (114.4 ± 7.3 mmHg vs. 101.3 ± 10.8 mmHg, *p* < 0.001). However, at T8, the MAP in the ciprofol group was lower than that in the propofol group (94.6 11.2 mmHg vs. 106.4 ± 17.3 mmHg, *p* < 0.001). This difference may be attributed to two cases (4%) in the ciprofol group and eight cases (15%) in the propofol group, where the MAP increased to more than 40% above the baseline value during tracheal extubation (Figure 2).

### 3.2 Rate of target MAP achievement during cross-clamping

During cross-clamping of the carotid artery (T4), the MAP was 17% higher than the baseline in the ciprofol group and 7% higher in the propofol group (*p* < 0.001) (Table 2). Despite a statistically significant difference in the target MAP achievement rates (38.0% vs. 17.0%, *p* = 0.017), the target MAP was reached in fewer than 50% of patients in both groups. Five minutes after cross-clamping (T0), the MAP was 26% higher than the baseline in the ciprofol group and 10% higher in the propofol group (*p* < 0.001). The rate of target MAP achievement, defined as a MAP at least 20% higher than the baseline MAP, significantly differed between the groups (76.0% vs. 20.8%, *p* < 0.001). Ten minutes after cross-clamping, the target MAP achievement rate was similar between the groups (80.0% vs. 64.2%, *p* = 0.074), indicating that in the early stage of carotid cross-clamping, the target MAP was reached faster in the ciprofol group than in the propofol group.

**Table 2:** MAP increase percentage and target achievement rate at different time points (T4, T5, and T6) during cross-clamping.

MAP	Overall, *N* = 103^a^	Ciprofol, *N* = 50^a^	Propofol, *N* = 53^a^	*p* value^b^
T4_T0	0.12 (0.12)	0.17 (0.08)	0.07 (0.13)	**<0.001**
T4_T0_YN				**0.017**
Yes	28 (27.2%)	19 (38.0%)	9 (17.0%)	
No	75 (72.8%)	31 (62.0%)	44 (83.0%)	
T5_T0	0.18 (0.14)	0.26 (0.10)	0.10 (0.12)	**<0.001**
T5_T0_YN				**<0.001**
Yes	49 (47.6%)	38 (76.0%)	11 (20.8%)	
No	54 (52.4%)	12 (24.0%)	42 (79.2%)	
T6_T0	0.25 (0.10)	0.27 (0.08)	0.23 (0.11)	0.173
T6_T0_YN				0.074
Yes	74 (71.8%)	40 (80.0%)	34 (64.2%)	
No	29 (28.2%)	10 (20.0%)	19 (35.8%)	

a Mean (SD); *n* (%).

b Wilcoxon rank sum test; Pearson’s Chi-squared test; Fisher’s exact test.

T0 (before anesthesia induction); T4 (during cross-clamping of the carotid artery); T5 (5 min after cross-clamping); T6 (10 min after cross-clamping).

Bold values indicate statistical significance (*p* < 0.05).

### 3.3 Medication dosage and timing and net fluid intake

The ciprofol group had higher MAPs and compliance rates during cross-clamping of the carotid artery but received less norepinephrine (403.5 μg vs. 610.0 μg, *p* < 0.001) (Table 3). Urapidil administration during extubation showed no significant intergroup difference (*p* = 0.574). Notably, 90.3% of patients (93/103) did not receive urapidil, with comparable non-administration rates between the ciprofol (48/50, 96%) and propofol (45/53, 84.9%) groups. For analytical purposes, non-administered cases were treated as 0 mg doses, while Table 3 maintains the original “missing” designation to preserve data transparency. There were no significant differences between the two groups regarding other drug dosages, surgical or anesthesia times, cross-clamping times, or net fluid intake.

**Table 3:** Comparison of drug dosages, surgical and anesthesia times, cross-clamping times, and net fluid intake.

Variable	Overall, *N* = 103^a^	Ciprofol, *N* = 50^a^	Propofol, *N* = 53^a^	*p* value^b^
Norepinephrine μg	524.0 (383.0, 663.5)	403.5 (331.3, 453.0)	610.0 (551.0, 757.0)	**<0.001**
Urapidil mg	12.5 (12.5, 12.5)	12.5 (12.5, 12.5)	12.5 (12.5, 15.6)	0.574
Missing	93	48	45	
Midazolam mg	3.0 (3.0, 4.0)	3.0 (2.0, 4.0)	3.0 (3.0, 4.0)	0.704
Sufentanil μg	35.0 (30.0, 35.0)	35.0 (30.0, 35.0)	35.0 (30.0, 35.0)	0.423
Remifentanil mg	1.5 (1.3, 1.8)	1.5 (1.3, 1.7)	1.5 (1.4, 1.9)	0.434
Cisatracurium mg	16.0 (15.5, 18.0)	16.0 (16.0, 18.0)	16.0 (15.0, 17.0)	0.478
Anesthesia-time min	169.0 (148.5, 185.5)	171.0 (149.5, 184.0)	166.0 (149.0, 186.0)	0.496
Surgery-time min	110.0 (98.0, 121.0)	112.0 (99.3, 121.8)	104.0 (98.0, 121.0)	0.301
Carotid cross clamping-time min	31.0 (27.0, 35.0)	31.0 (27.0, 34.0)	32.0 (27.0, 40.0)	0.163
Net fluid intake ml	640.0 (455.0, 820.0)	675.0 (515.0, 825.0)	635.0 (415.0, 910.0)	0.402

a Median (IQR).

b Wilcoxon rank sum test.

Bold values indicate statistical significance (*p* < 0.05).

### 3.4 Postoperative complications

Four patients (8.0%) in the ciprofol group and 13 patients (24.5%) in the propofol group experienced eCD (*p* = 0.024) (Table 4). With respect to the incidence of other complications, including headache, no clear significant differences were observed. Within 30 days, one patient in the ciprofol group experienced a stroke, and one patient died; however, in the propofol group, one patient experienced a stroke, and another experienced a myocardial infarction. However, there is currently no evidence to suggest that these events are related to the intraoperative medications administered.

**Table 4:** Comparison of postoperative complications between the ciprofol and propofol groups.

Variable	Overall *N* = 103^a^	Ciprofol *N* = 50^a^	Propofol *N* = 53^a^	Ciprofol vs. propofol ARD (95% CI)^b^	*p* value^c^
eCD				**−16.5% [−30.3, −2.7%]**	**0.024**
Yes	17 (16.5%)	4 (8.0%)	13 (24.5%)		
No	86 (83.5%)	46 (92.0%)	40 (75.5%)		
Headache				−11.2% [−21.6, 0.8%]	0.061
Yes	8 (7.8%)	1 (2.0%)	7 (13.2%)		
No	95 (92.2%)	49 (98.0%)	46 (86.8%)		
Stroke				0.1% [−5.2, 5.4%]	>0.999
Yes	2 (1.9%)	1 (2.0%)	1 (1.9%)		
No	101 (98.1%)	49 (98.0%)	52 (98.1%)		
MI				−1.9% [−5.6, 1.8%]	>0.999
Yes	1 (1.0%)	0 (0.0%)	1 (1.9%)		
No	102 (99.0%)	50 (100.0%)	52 (98.1%)		
Death				2.0% [−1.8, 5.8%]	0.485
Yes	1 (1.0%)	1 (2.0%)	0 (0.0%)		
No	102 (99.0%)	49 (98.0%)	53 (100.0%)		

a *n* (%).

b Newcombe–Wilson.

c Pearson’s Chi-squared test; Fisher’s exact test.

ARD, Absolute Risk Difference; eCD, early cognitive dysfunction; MI, myocardial Infarction.

Bold values indicate statistical significance (*p* < 0.05).

### 3.5 Confounding factors

Results of the multivariable analysis for norepinephrine using a linear regression model (Table 5).

**Table 5:** Results of multivariable analysis of results for norepinephrine using a linear regression model.

Dependent: norepinephrine μg	Unit	Value	Coefficient (multivariable)
Age			
[52.0, 89.0]	Mean (SD)	530.9 (199.7)	1.94 (−2.72 to 6.59, *p* = 0.410)
BMI			
[19.0, 30.0]	Mean (SD)	530.9 (199.7)	**−15.52 (−29.27 to −1.77, *p* = 0.027)**
ASA			
II	Mean (SD)	496.8 (180.4)	–
III	Mean (SD)	545.6 (207.0)	35.35 (−41.31 to 112.00, *p* = 0.362)
Hypertension			
No	Mean (SD)	506.0 (193.6)	–
Yes	Mean (SD)	548.8 (203.7)	4.52 (−61.16 to 70.20, *p* = 0.892)
CHD			
No	Mean (SD)	509.0 (184.5)	–
Yes	Mean (SD)	575.4 (223.8)	29.17 (−44.29 to 102.62, *p* = 0.433)
Group			
Propofol	Mean (SD)	643.8 (191.0)	–
Ciprofol	Mean (SD)	411.3 (126.4)	**−226.97 (−291.74 to −162.21, *p* < 0.001)**

BMI, body mass index; ASA, American Society of Anesthesiologist; CHD, Coronary Heart Disease.

After adjusting for the other variables in the model, Group and BMI were significant.

Bold values indicate statistical significance (*p* < 0.05).

Linear Regression Model: Norepinephrine = Age + BMI + ASA + Hypertension + CHD + Group.

Number in dataframe = 103, Number in model = 103, Missing = 0, Log likelihood = −666.31, AIC = 1348.6, *R*-squared = 0.38, Adjusted *R*-squared = 0.35.

After adjusting for the other variables in the model, group and BMI were significant.

The amount of norepinephrine administered to the patients in the ciprofol group was 226.97 μg (95% CI −291.74 to −162.21) less than that administered to the patients in the propofol group. When the BMI increased by 1, the norepinephrine dosage decreased by 15.52 μg (95% CI −29.27 to −1.77).

## 4 Discussion

**Figure 3:**
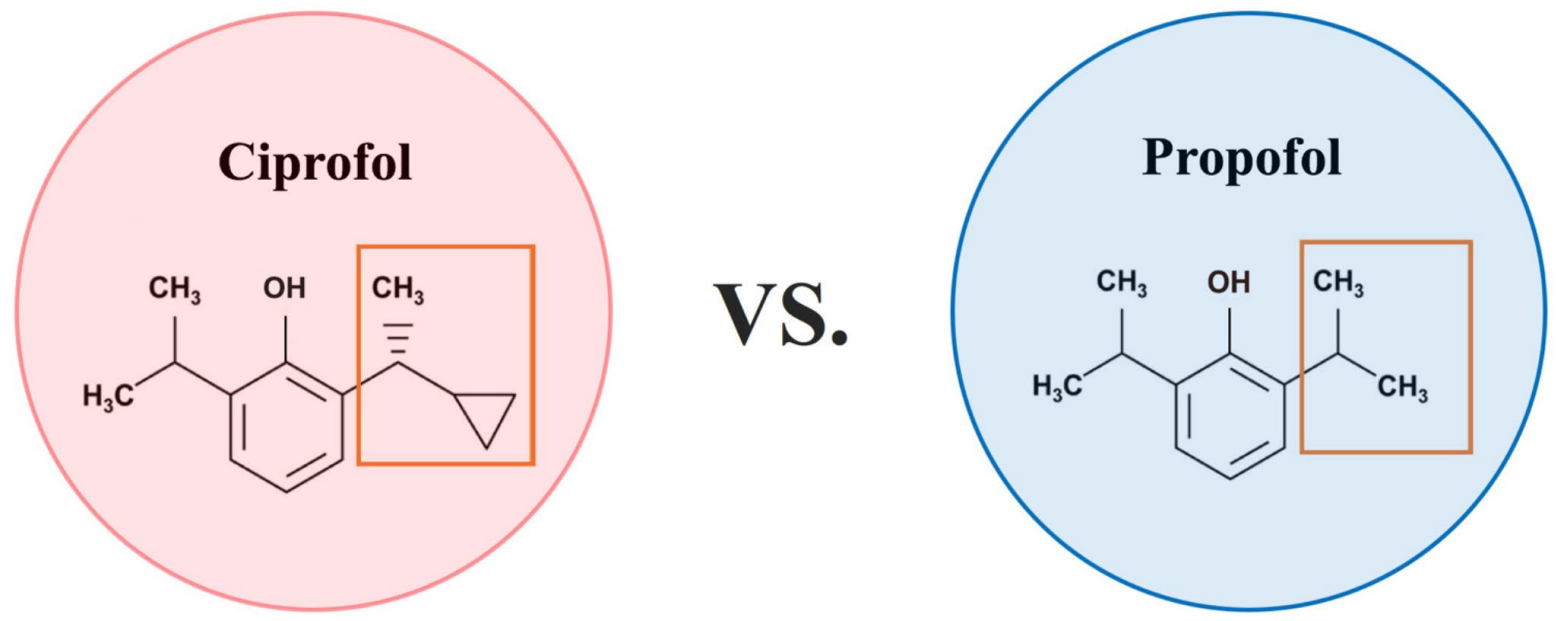
Comparison chart of ciprofol and propofol. Propofol and ciprofol are both phenolic compounds characterized by the presence of a benzene ring and a hydroxyl group. Ciprofol ([R]-2-[1-cyclopropylethyl]-6-isopropylphenol) retains the core structural framework of propofol, with the key modification being the replacement of an isopropyl substituent by a 1-cyclopropylethyl group.

Ciprofol and propofol are both *γ*-aminobutyric acid (GABA) receptor agonists and have dose-dependent effects. However, subtle structural differences between these compounds determine their pharmacological properties and clinical manifestations. Specifically, Propofol and ciprofol are both phenolic compounds characterized by the presence of a benzene ring and a hydroxyl group. Ciprofol ([R]-2-[1-cyclopropylethyl]-6-isopropylphenol) retains the core structural framework of propofol, with the key modification being the replacement of an isopropyl substituent by a 1-cyclopropylethyl group (Figure 3). This modification enhances the spatial effect of ciprofol, significantly increasing its affinity for the GABAA receptor. As a result, ciprofol is 4–5 times more potent than propofol (17). Studies have shown that the brain tissue concentration of ciprofol is 3.2-fold greater than that in plasma, indicating its ability to cross the blood–brain barrier and produce central inhibitory effects (18). The higher lipophilicity of ciprofol enables it to enter cell membranes quickly, which lowers the concentration of free molecules in the emulsion. These properties reduce drug side effects and their impact on the circulatory system. This high lipophilicity also promotes rapid onset and metabolism, leading to a shorter half-life and a higher pharmacokinetic clearance rate (19, 20). Pharmacokinetic studies have shown that ciprofol has a large volume of distribution (Vss > 7.79 L/kg) and rapid clearance (15.7 L/kg /h in rats), along with a short half-life (0.72 h in rats, 1.44 h in beagle dogs), supporting its rapid onset and fast metabolic properties, which contribute to maintaining hemodynamic stability during surgery (18). The rapid activation and metabolism of the anesthetics can compensate for rapid changes in the MAP during surgery, meeting the specific circulatory requirements during CEA.

Many studies have revealed that intravenous ciprofol (0.4 mg/kg) has similar sedative effects but lower risks of anesthesia-related adverse reactions than propofol (2.0 mg/kg) does (17, 21). Another meta-analysis of 12 randomized controlled trials involving nonpediatric and nonelderly patients aged 34–58 years revealed that ciprofol significantly reduced the incidences of injection pain and hypotension (22). However, current studies on the efficacy and safety of ciprofol have primarily focused on relatively healthy adult populations. There has been limited investigation in higher-risk surgical cohorts, including only a handful of procedures such as cardiac transcatheter aortic valve replacement (TAVR) (23). Furthermore, evidence remains scarce regarding its potential benefits in neurosurgical procedures, particularly those requiring careful management of cerebral perfusion like CEA. Patients with carotid stenosis, due to narrowing of the major cerebral blood-supplying arteries, experience dilation of collateral microarteries and capillaries. Over time, this leads to impaired smooth muscle contraction function and reduced vascular resistance, causing cerebral blood flow to fluctuate dramatically beyond the normal range in response to changes in the MAP. The baroreceptor sensitivity of the carotid sinus is further impaired due to the compressive behavior of plaques and injury during surgery at the site of stenosis (24). Patients with impaired cerebral vasoregulation cannot withstand rapid changes in MAP, and dramatic fluctuations in cerebral blood flow are directly associated with increased risks of postoperative complications and mortality. Intraoperative hypotension is associated with eCD, stroke, and cardiovascular events, whereas hypertension may lead to complications such as cerebral hyperperfusion syndrome and stroke (8, 25). Therefore, CEA requires very strict intraoperative circulatory management (26). In this study cohort, although the target MAP achievement rate during the initial phase of carotid cross-clamping (T4) did not exceed 50% in either group, the ciprofol group demonstrated a significant advantage at 5 min post-clamping (T5), with 76% of patients achieving the target MAP compared to only 21% in the propofol group. The basic demographic characteristics of the patients were similar between the two groups, and we emphasize that the standardized protocol for concomitant anesthetics and fluid management minimized their potential as confounding variables, allowing the differences between ciprofol and propofol to be more clearly isolated. This finding clearly indicates that ciprofol facilitates faster hemodynamic optimization during the critical early phase of carotid cross-clamping. Pharmacologically, the unique structural features and rapid metabolic properties of ciprofol may provide a plausible explanation for its superior hemodynamic stability (17, 19). Clinical evidence underscores the importance of maintaining adequate cerebral perfusion pressure during carotid cross-clamping to prevent cerebral ischemia (27). The earlier achievement of the target MAP with ciprofol may theoretically reduce the duration of cerebral hypoperfusion, thereby potentially mitigating ischemic injury. This early hemodynamic optimization may cumulatively reduce intraoperative ischemia–reperfusion injury and microcirculatory disturbances, laying the foundation for reducing long-term cerebrovascular risks. This mechanistic hypothesis aligns with our observations: the incidence of eCD was significantly lower in the ciprofol group (8.0% vs. 24.5%, *p* = 0.024). Previous studies also support the association between inadequate MAP during cross-clamping and postoperative neurocognitive complications (4, 6). However, it is important to note that while the temporal correlation between improved MAP control and reduced eCD risk is evident, a causal relationship requires further investigation. As a multifactorial clinical endpoint, eCD may also involve other contributing factors such as intraoperative microemboli and individual variability in cerebrovascular reserve (28). Thus, while affirming the hemodynamic advantages of ciprofol, cautious interpretation of its impact on neurocognitive outcomes is warranted. Although no significant differences were observed in the 30-day stroke, myocardial infarction, or mortality rates between groups, the small sample size may have limited the statistical power of this study. Notably, the quality of intraoperative hemodynamic management has been linked to long-term neurological outcomes in carotid surgery patients (29, 30), suggesting that the short-term benefits of ciprofol may have biological plausibility for translating into long-term clinical advantages. While our study highlights the intraoperative hemodynamic superiority of ciprofol as a foundation for short-term outcome improvement, its long-term benefits require validation through larger-scale, multicenter studies with extended follow-up, combined with deeper exploration of pathophysiological mechanisms.

We further observed that while the propofol group had a slower target MAP achievement rate during carotid artery cross-clamping, they had a significantly higher rate of hypertension during tracheal extubation. Specifically, eight (15%) patients had a MAP more than 40% higher than the baseline, a factor that may contribute to postoperative headaches (31). Further exploration and confirmation of the correlation between intraoperative MAP variability and headache caused by cerebral hyperperfusion syndrome are needed in future studies. The mechanism underlying the higher incidence of hypertension in the propofol group during tracheal extubation remains uncertain, although we hypothesize it may be associated with the significantly higher doses of norepinephrine required in this group (403.5 μg vs. 610.0 μg, *p* < 0.001). Several factors may influence norepinephrine requirements. To account for potential confounders, a multivariable linear regression model was constructed, which confirmed that after adjusting for age, ASA status, hypertension, and CHD, the anesthetic group remained a significant predictor of norepinephrine dosage. Notably, norepinephrine, an adrenergic receptor agonist, primarily activates *α* receptors (nonselective for *α*1 and *α*2) and weakly activates *β*1 receptors. Through the cAMP/PKA and ERK pathways, it synergistically activates eNOS (endothelial nitric oxide synthase) under acute conditions, leading to endothelial dysfunction and affecting vascular elasticity and stiffness. The duration of these effects is related to specific pathological conditions, oxidative stress levels, and intraoperative ischemia–reperfusion injury (32). Decreased vascular compliance and increased resistance during the perioperative period increase the MAP, especially in patients with impaired cerebral vascular autoregulation, who are more sensitive to changes in MAP (33, 34).

Additionally, the regression analysis also identified that the dosage of norepinephrine decreased with increasing BMI, confirming it as an independent factor influencing requirements. Several studies have confirmed this (35). Although the distribution of baseline BMI was well balanced between the ciprofol and propofol groups [24.5 (22.3, 26.0) vs. 25.0 (23.0, 26.0), *p* = 0.479], indicating that there was no significant difference in BMI between the two groups, further analysis revealed that BMI was an independent factor influencing norepinephrine dosage in both groups. This suggests that while BMI did not confound the primary outcome between the groups, it still played a role in determining the individual norepinephrine requirements within each group. Therefore, the balanced distribution of BMI across groups ensured that this factor did not bias the overall comparison of norepinephrine demand between patients administered ciprofol and those administered propofol, but its independent effect on dosage was still accounted for in the regression model. The administration of norepinephrine should be improved in the future to reduce the risk of postoperative adverse events, and better anesthesia management strategies are needed for patients undergoing CEA under ciprofol anesthesia.

Based on our findings, we propose the following clinical recommendations. Ciprofol may be considered a favorable anesthetic option for patients undergoing CEA, as it not only mitigates blood pressure reduction but also facilitates a rapid and targeted elevation of MAP to meet the specific hemodynamic demands during carotid cross-clamping. This proactive blood pressure management aligns more closely with the rigorous hemodynamic requirements of CEA. Furthermore, the use of ciprofol reduces norepinephrine requirements and shows a correlative association with a decreased incidence of eCD. Nevertheless, regardless of the anesthetic selected, stringent individualized hemodynamic monitoring, including continuous arterial blood pressure and cerebral oximetry, remains essential throughout all phases of CEA to promptly identify and manage deviations from the target blood pressure range. It should be noted that no intravenous anesthetic is perfect, and balancing anesthetic efficacy with hemodynamic demands during CEA remains a clinical challenge. Our study aims to contribute to the identification of more optimal anesthetic strategies for specific patient populations undergoing this procedure.

## 5 Limitations

As a single-center retrospective study, our findings may be influenced by unmeasured confounders and selection bias. Although we excluded patients who deviated from standardized anesthesia protocols to ensure internal validity, this restriction may limit the generalizability of our results to centers with different practice standards. Future multicenter prospective studies should adopt broader inclusion criteria and standardized data collection protocols to validate our conclusions.

Additionally, the exclusion of 14 patients due to missing intraoperative monitoring data or unavailable postoperative complication data may introduce bias. To address this, we performed a sensitivity analysis comparing baseline characteristics between included and excluded patients, which revealed no significant differences in age, sex, ASA status or any other baseline variables (all *p* > 0.05). Nevertheless, the potential influence of unmeasured variables (e.g., socioeconomic factors) cannot be entirely ruled out.

## 6 Conclusion

In this study comparing ciprofol and propofol for carotid endarterectomy, ciprofol demonstrated superior hemodynamic stability, with a significantly higher rate of target MAP achievement during early cross-clamping and reduced norepinephrine requirements. Additionally, the ciprofol group showed a lower incidence of eCD. These findings support ciprofol as a preferable anesthetic for CEA, offering improved blood pressure control, minimized vasopressor dependence, and enhanced postoperative safety.

## Data Availability

The original contributions presented in the study are included in the article/Supplementary material, further inquiries can be directed to the corresponding author/s.
