# Supplementary material for: Comparative study of ciprofol vs. propofol in carotid endarterectomy: focusing on mean arterial pressure, vasoactive drug use, and postoperative complications
Source: Front Med (Lausanne). 2025 Oct 17;12:1648417. doi: 10.3389/fmed.2025.1648417 (PMC12575303; doi:10.3389/fmed.2025.1648417)
Supplement: Supplementary file 1 [file Table_1.docx]

**Supplemental Table 1. Comparison of Baseline Characteristics Between Included and Excluded Patients**

| **Characteristic** | **Overall**, N = 117^a^ | **Included***,* N = 103^a^ | **Excluded***,* N = 14^a^ | ***p* value**^b^ |
| --- | --- | --- | --- | --- |
| **Age** | 71.0 (66.0, 75.0) | 71.0 (66.0, 75.0) | 72.0 (67.0, 76.0) | 0.450 |
| **Sex** |  |  |  | 0.820 |
| Female | 32 (27.4%) | 29 (28.2%) | 3 (21.4%) |  |
| Male | 85 (72.6%) | 74 (71.8%) | 11 (78.6%) |  |
| **BMI** | 25.0 (23.0, 26.0) | 25.0 (23.0, 26.0) | 24.5 (22.0, 26.5) | 0.650 |
| **ASA** |  |  |  | 0.520 |
| II | 34 (29.1%) | 31 (30.1%) | 3 (21.4%) |  |
| III | 83 (70.9%) | 72 (69.9%) | 11 (78.6%) |  |
| **Smoke** |  |  |  | 0.730 |
| Yes | 45 (38.5%) | 41 (39.8%) | 4 (28.6%) |  |
| No | 72 (61.5%) | 62 (60.2%) | 10 (71.4%) |  |
| **Hypertension** |  |  |  | 0.880 |
| Yes | 68 (58.1%) | 60 (58.3%) | 8 (57.1%) |  |
| No | 49 (41.9%) | 43 (41.7%) | 6 (42.9%) |  |
| **DM** |  |  |  | 0.610 |
| Yes | 56 (47.9%) | 50 (48.5%) | 6 (42.9%) |  |
| No | 61 (52.1%) | 53 (51.5%) | 8 (57.1%) |  |
| **CHD** |  |  |  | 0.420 |
| Yes | 38 (32.5%) | 34 (33.0%) | 4 (28.6%) |  |
| No | 79 (67.5%) | 69 (67.0%) | 10 (71.4%) |  |
| **CVA history** |  |  |  | 0.990 |
| Yes | 35 (29.9%) | 31 (30.1%) | 4 (28.6%) |  |
| No | 82 (70.1%) | 72 (69.9%) | 10 (71.4%) |  |

^a^Median (IQR); n (%)

^b^Wilcoxon rank sum test; Pearson's Chi-squared test

Abbreviation: BMI, body mass index; ASA, American Society of Anesthesiologist; DM, Diabetes Mellitus; CHD, Coronary Heart Disease; CVA history, history of cerebrovascular accident
